# Using Infodemiology Metrics to Assess Public Interest in Liver Transplantation: Google Trends Analysis

**DOI:** 10.2196/21656

**Published:** 2021-08-17

**Authors:** Maria Effenberger, Andreas Kronbichler, Erica Bettac, Felix Grabherr, Christoph Grander, Timon Erik Adolph, Gert Mayer, Heinz Zoller, Paul Perco, Herbert Tilg

**Affiliations:** 1 Department of Internal Medicine I, Gastroenterology, Hepatology, Endocrinology and Metabolism Medical University of Innsbruck Innsbruck Austria; 2 Department of Internal Medicine IV, Nephrology and Hypertensiology Medical University of Innsbruck Innsbruck Austria; 3 Department of Psychology Washington State University Vancouver Vancouver, WA United States

**Keywords:** digital medicine, search trends, public awareness, infodemiology, eHealth

## Abstract

**Background:**

Liver transplantation (LT) is the only curative treatment for end-stage liver disease. Less than 10% of global transplantation needs are met worldwide, and the need for LT is still increasing. The death rates on the waiting list remain too high.

**Objective:**

It is, therefore, critical to raise awareness among the public and health care providers and in turn increasingly acquire donors.

**Methods:**

We performed a Google Trends search using the search terms *liver transplantation* and *liver transplant* on October 15, 2020. On the basis of the resulting monthly data, the annual average Google Trends indices were calculated for the years 2004 to 2018. We not only investigated the trend worldwide but also used data from the United Network for Organ Sharing (UNOS), Spain, and Eurotransplant. Using pairwise Spearman correlations, Google Trends indices were examined over time and compared with the total number of liver transplants retrieved from the respective official websites of UNOS, the Organización Nacional de Trasplantes, and Eurotransplant.

**Results:**

From 2004 to 2018, there was a significant decrease in the worldwide Google Trends index from 78.2 in 2004 to 20.5 in 2018 (–71.2%). This trend was more evident in UNOS than in the Eurotransplant group. In the same period, the number of transplanted livers increased worldwide. The waiting list mortality rate was 31% for Eurotransplant and 29% for UNOS. However, in Spain, where there are excellent awareness programs, the Google Trends index remained stable over the years with comparable, increasing LT numbers but a significantly lower waiting list mortality (15%).

**Conclusions:**

Public awareness in LT has decreased significantly over the past two decades. Therefore, novel awareness programs should be initialized.

## Introduction

### Background

Liver transplantation (LT) remains to be the only curative therapy for patients affected by end-stage liver disease, cirrhosis with hepatocellular carcinoma, acute fulminant hepatic failure, hepatocellular carcinoma, hilar cholangiocarcinoma, and several metabolic disorders [1,2].

Each year, approximately 12,000 LTs are performed in Europe and the United States, with numbers significantly increasing over time [3]. At present, more than 70% of liver transplant recipients survive for at least 5 years, compared with 20% in the 1980s. Such statistics are especially encouraging, considering that transplanted patients tend to have more severe diseases [4]. Several factors increase the survival of patients with LT, including better control of disease before LT, improved surgical techniques and surgeons specialized in these techniques, improved organ preservation, and advanced immunosuppressive therapy regimens [5]. However, the improved success rate of LT has resulted in substantial organ shortages [4]. Such shortages have led to a prolonged time for patients on the waiting list and increased waiting list mortality [6-8].

In 2017, the median pretransplant waiting time among active waitlisted adults was 10 months in the Eurotransplant region and approximately 9 months in the United States [9]. Mortality on the list was 18.7% in the Eurotransplant region, which is comparable with the United States’ 19.8% of listed patient deaths before transplantation. It is important to note that limited donor organs are available from deceased donors after brain death [6].

The discrepancy between available liver allografts and transplant candidates continues to increase globally. Significant efforts have been made to raise the donor pool in Europe and the United States, such as using extended criteria donor organs [10], inventing extracorporeal normothermic or hypothermic organ perfusion systems [11], and accepting liver allografts as donation after circulatory determination of death (DCDD) [12]. Despite these efforts, there remains no significant decrease in waiting list mortality. To close the gap between available organs and the number of patients in need of LT, a higher awareness and acceptance of the transplant and donor program in the general population, as well as among health care providers, is a potentially effective strategy.

Infodemiology is an emerging area of research among health informatics, health care professionals, and patients. Introduced in 2002 [13], the term infodemiology is defined as a new area of scientific research that holds great promise for improving public health by focusing on specific internet searches for user-contributed health-related content [13-15]. These searches track public opinion, behavior, attention, knowledge, and attitudes [16].

### Objectives

The first study indicated a correlation between searches on the internet and incidence in the field of *infectious diseases* [17]. The number of infodemiological studies has increased over the past decade, and these studies have used Twitter and Google [18]. Many researchers have used the infodemiological approach to study various health-related topics, for example, infectious diseases such as influenza or HIV/AIDS, chronic diseases such as multiple sclerosis, or patterns of smoking and tobacco use [19-27]. A particular interest in infodemiology has risen because of the ongoing COVID-19 pandemic [28], as research informs about the speed of misinformation [29], correlation between search behavior and COVID-19 related mortality [30], mental health issues [31], and pressing health care topics such as telehealth capacity of hospitals [32]. In addition, these data are becoming valuable tools for exploring human behavior. The advantage of infodemiology is that metrics are available in real time, which can provide quantitative and qualitative data while being automatically and inexpensively collected.

The analysis of internet search queries offers information on the extent of public attention, thereby reflecting the level of public awareness [33-36]. Google Trends is one of the most widely used tools for this purpose. It is not only used to study public interest in health care topics but also to predict disease occurrence and outbreaks [17,37,38].

In this study, we evaluated public interest in LT over time using Google Trends data and compared them with the number of transplanted livers reported from the United Network for Organ Sharing (UNOS), the Organización Nacional de Trasplantes (ONT), and the Eurotransplant regions.

## Methods

### Retrieving Transplantation Numbers for UNOS, ONT, and Eurotransplant

Data were retrieved by accessing the respective websites of the transplant organizations UNOS, ONT, and Eurotransplant [39-41]. We extracted information on living and deceased donors over a period of 15 years (2004-2018) for the following countries: the United States (UNOS), Spain (ONT), Austria, Belgium, Croatia, Germany, Hungary, Luxembourg, and the Netherlands (belonging to the Eurotransplant countries). No organs from executed prisoners were used in these transplant organizations.

### Retrieving Google Trends Data on LT

The Google Trends tool was used on October 15, 2020, to retrieve data on internet user search activities in the context of LT [42]. Worldwide Google Trends indices were retrieved from January 2004 onward using the search terms, *liver transplantation* and *liver transplant*. We retrieved Google Trends indices for the United States, Spain, and European countries, in part included in the Eurotransplant network, namely Austria, Belgium, Croatia, Germany, Hungary, and the Netherlands. No Google Trends indices could be retrieved for Luxembourg and Slovenia. Whereas the worldwide search was performed in English, individual searches across non–English-speaking countries were performed in their respective official languages. We used individual search terms and combined the search terms yielding Google Trends results in larger queries, as listed in Table 1. On the basis of monthly data, annual average Google Trends indices were calculated for the years 2004 to 2018 and used to generate the line plots with the ggplot2 package of the statistical software R (version 3.4.1; R Foundation for Statistical Computing). It is important to note that none of the queries in the Google database for this study can be associated with a particular individual. The database does not retain information about the identity, IP address, or specific physical location of any user. The Spearman correlation coefficient was used to determine pairwise correlations between total liver transplant numbers per country and Google Trends indices.

**Table 1:** Google Trends search query listing (2004-present).

Region	Language	Google Trends search query
Worldwide	English	*liver transplantation* and *liver transplant*
United States	English	*liver transplantation* and *liver transplant*
Spain	Spanish	*trasplante de hígado*, *trasplante higado*, and *trasplante de higado*
Belgium	French	*transplantation hépatique* and *levertransplantatie*
The Netherlands	Dutch	*Levertransplantatie*
Germany	German	*Lebertransplantation*
Austria	German	*Lebertransplantation*
Hungary	Hungarian	*májátültetés* and *májtranszplantáció*
Croatia	Croatian	*transplantacija jetre*

## Results

### Google Trends and Trends for LT Worldwide

**Figure 1:**
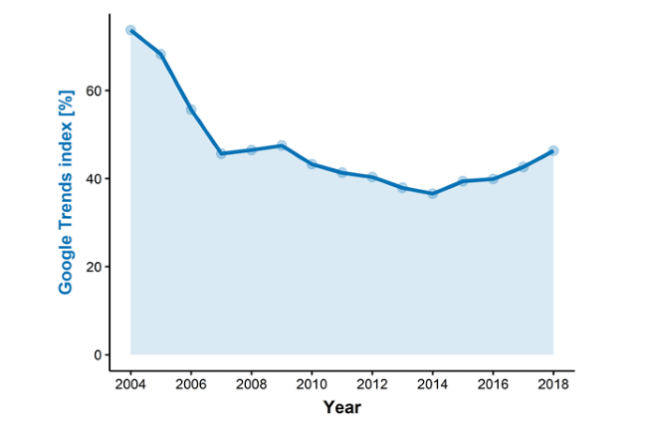
Worldwide interest in liver transplantation using Google Trends.

The global Google Trends index for LT decreased from 73.8 to 36.6 (–50.4%) between 2004 and 2014. In 2018, there was a slight upward trend in the LT index to 46.3 (+27.5%; Figure 1).

**Figure 2:**
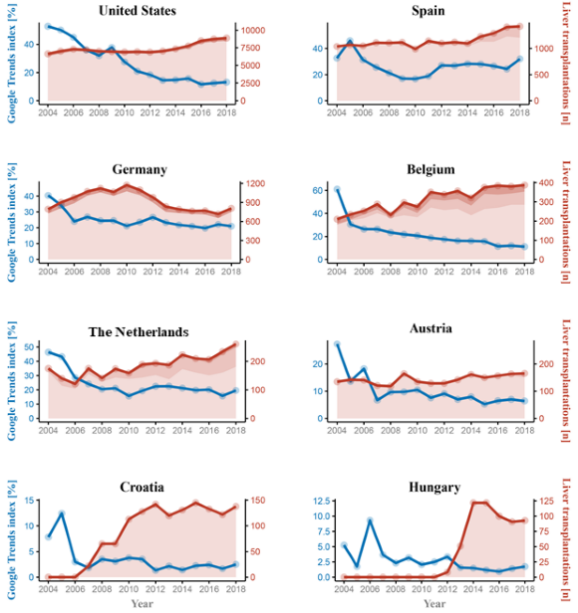
Google Trends and number of liver transplants in Eurotransplant, United Network for Organ Sharing, and Organización Nacional de Trasplantes over time.

A similar trend was observed for the UNOS, with the Google Trends index dropping from 59.2 to 38.8 (–34.5%) in 2014 and an upward trend since then to 50.3 (+46.2%) until 2018. Similarly, Google Trends indices in the Eurotransplant region exhibited a decline in numbers in all Eurotransplant countries across the same period (Figure 2; Tables 2 and 3).

**Table 2:** The respective year, number of search queries using Google Trends, and the total number of liver transplantations performed in different countries are provided (deceased donor and living donor).

Year	World Google Trends index	United States Google Trends	United States TX^a^ total	Spain Google Trends index	Spain TX total	Belgium Google Trends index	Belgium TX total	Luxembourg TX total	The Netherlands Google Trends index	The Netherlands TX total
2004	73.8	59.1667	6642	8.3	1040	22.8	210	1	19.7	175
2005	68.3	60.5833	7015	24.1	1070	11.9	234	2	5.3	140
2006	55.7	49.3333	7302	12.2	1051	16.3	253	5	6.4	121
2007	45.7	45.9167	7202	6.0	1112	8.6	288	1	5.7	175
2008	46.5	45.25	7000	10.3	1108	13.4	232	0	5.5	141
2009	47.5	52.0833	6958	6.3	1119	16.6	296	0	10.8	174
2010	43.3	43.3333	6893	8.9	989	10.4	275	3	8.0	159
2011	41.3	41.8333	6931	7.6	1145	12.5	351	9	8.6	189
2012	40.3	40.0833	6876	13.4	1101	10.1	339	4	11.5	193
2013	37.9	38.1667	7026	13.8	1122	12.3	357	6	8.1	187
2014	36.6	38.0833	7344	13.3	1100	10.3	321	3	9.7	223
2015	39.4	39.9167	7775	17.7	1229	11.3	375	3	9.3	210
2016	39.9	39.75	8497	15.3	1292	11.4	384	3	9.3	207
2017	42.7	43.9167	8740	14.3	1413	9.8	380	9	8.1	234
2018	46.3	50.25	8875	17.3	1426	11.8	387	7	9.0	261

^a^TX: number of transplantations.

**Table 3:** The respective year, number of search queries using Google Trends, and the total number of liver transplantations performed are provided (deceased donor and living donor).

Year	Germany Google Trends index	Germany TX^a^ total	Austria Google Trends index	Austria TX total	Slovenia TX total	Hungary Google Trends index	Hungary TX total	Croatia Google Trends index	Croatia TX total
2004	61.6	795	15.5	135	24	0.0	0	8.3	0
2005	52.0	901	14.6	142	15	0.0	0	8.3	0
2006	35.3	979	6.9	141	21	8.3	0	0.0	0
2007	32.6	1074	16.2	121	15	1.4	0	0.8	22
2008	35.8	1122	11.7	119	22	1.0	0	1.5	65
2009	35.3	1065	9.4	165	22	2.2	0	2.0	65
2010	29.2	1173	8.8	135	34	0.6	0	1.7	113
2011	33.6	1097	11.8	129	24	2.2	0	1.3	128
2012	36.3	980	12.9	129	38	1.8	8	1.2	142
2013	31.8	836	9.8	142	35	1.3	51	1.9	120
2014	30.1	793	9.3	162	34	1.2	122	1.0	131
2015	27.9	765	5.6	150	43	1.3	122	2.3	145
2016	27.3	771	7.3	157	37	0.8	100	1.3	133
2017	30.3	716	11.2	164	34	1.3	91	1.8	122
2018	27.3	807	6.5	166	29	1.9	93	2.3	138

^a^TX: number of transplantations.

**Figure 3:**
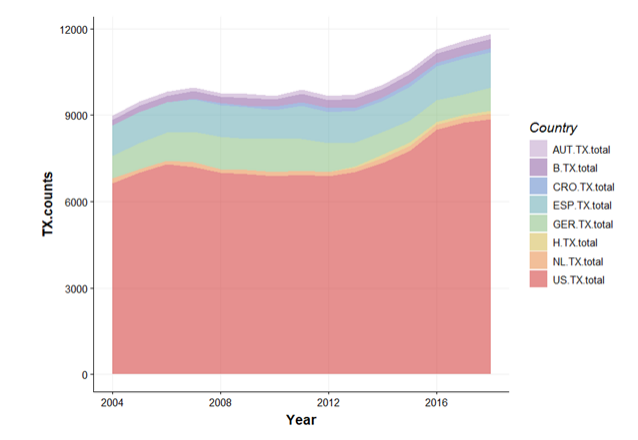
Number of liver transplants in Eurotransplant, United Network for Organ Sharing, and Organización Nacional de Trasplantes. AUT: Austria; B: Belgium; CRO: Croatia; ESP: Spain; GER: Germany; GT: Google Trends; H: Hungary; NL: the Netherlands; TX: nuber of transplantations; US: United States.

In the same period (2004-2018), UNOS reported the most significant increase in deceased donor liver transplants from 6642 to 8875 (+34.0%). Conversely, the number of living donor donations remained stable during the same period. The number of LTs increased by 24.0% and 18.2% in the Eurotransplant and ONT, respectively (Figure 3; Multimedia Appendix 1), and the number of (both deceased and live donor) LTs increased by 24.0% and 18.2% in the Eurotransplant and ONT, respectively (Figure 3; Multimedia Appendix 1).

**Figure 4:**
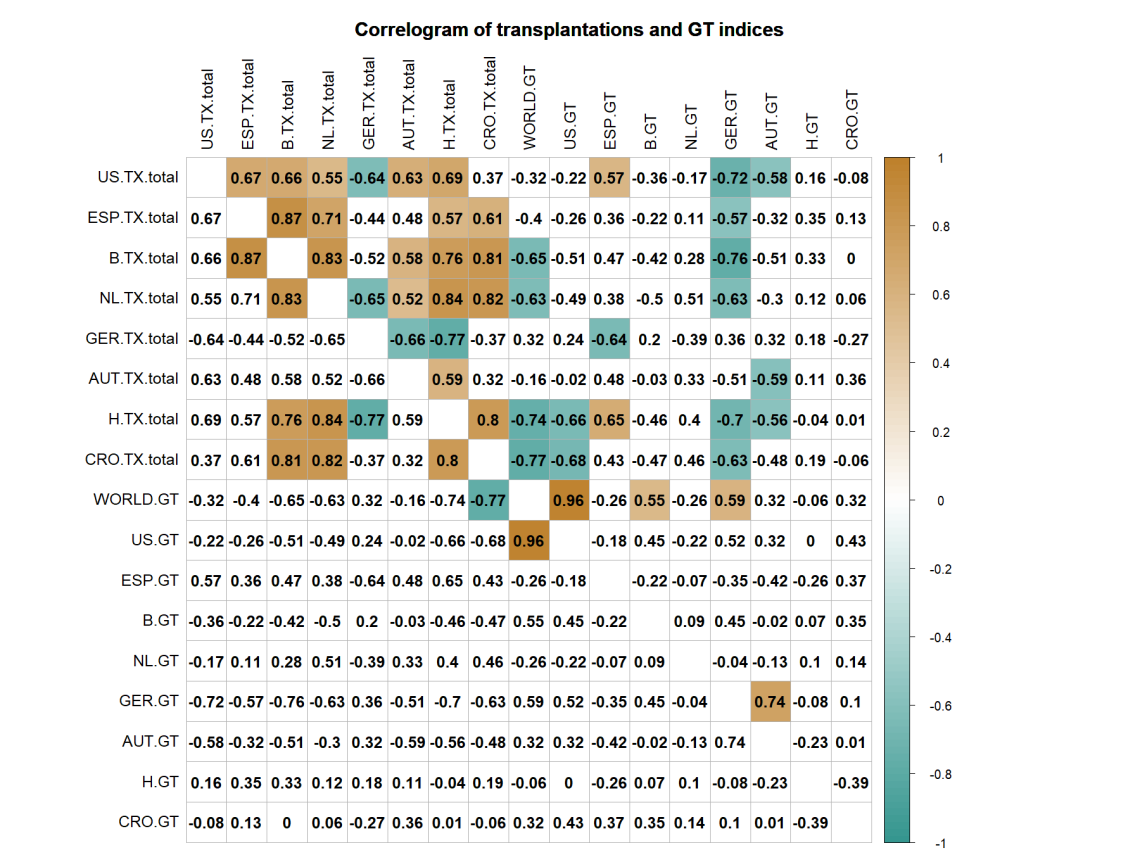
Correlogram of total transplant numbers and Google Trends indices of investigated countries. Correlations are based on the Spearman correlation coefficient. Pairwise correlations between total transplant numbers per country and Google Trends indices were calculated. Significant correlations with *P* values <.05 and <.001 are highlighted by a colorful background in the upper and lower half of the matrix, respectively. AUT: Austria; B: Belgium; CRO: Croatia; ESP: Spain; GER: Germany; GT: Google Trends; H: Hungary; NL: the Netherlands; TX: nuber of transplantations; US: United States.

Belgium and the Netherlands were the only 2 countries in the Eurotransplant region with a mild increase in living donor LT; however, in these countries, a significant decrease in the Google Trends index was observed (Belgium: –48.3%; the Netherlands: –53.3%; Multimedia Appendix 2). Similar downward trends were observed in all Eurotransplant countries. A correlogram of the total transplant numbers and Google Trends indices of the investigated countries are depicted in Figure 4**.** Most notably, even in Croatia, a country with 42 transplantations per million and a dissent solution, the Google Trends index significantly decreased from 7.8 to 2.5 (–75.7%). Google Trends changes and the number of transplants (deceased donor and living donor transplantations) in the respective countries over time are displayed in Figure 2. The number of DCDD donors in the Eurotransplant region and the UNOS area showed a mild increase. In 2018, only 8.08% (145/1795) and 5.71% (537/9412) of deceased donors were DCDD donors for LT in the Eurotransplant and UNOS regions, respectively (Multimedia Appendix 3).

### Google Trends and Waiting List Mortality

The waiting list mortality did not change significantly in UNOS (–5.2%) and Eurotransplant (–6.2%; Multimedia Appendix 4) regions. Even in Germany, the Eurotransplant country with the highest waiting list mortality (451/1379, 32.7%), the Google Trends index decreased from 40.4 to 21.1 (–48.5%). Furthermore, we analyzed the data of UNOS based on ethnicity. With no significant change over time, Hispanic individuals (2.4%), and American Indians and Alaska Natives (each 4.8%), had a significantly higher mortality on the waiting list than that of all ethnicities (Multimedia Appendix 5). An overview of changes in Google Trends over time, number of transplants (deceased donor, DCDD, and living donor transplantation) in the respective countries is depicted in Figure 3.

### Google Trends and LT Program in Spain

Spain exhibited a distinct Google Trends index pattern compared to other countries. The index slightly decreased until 2011 (the year of implementation of the DCDD program in Spain). A campaign for DCDD donors in the public, as well as in hospitals where potential donors are hospitalized, resulted in an increase in the Google Trends index. In the period of 15 years, we could not find a significant decrease in the Google Trends index (–1.8%). In fact, the number of transplanted livers increased because of DCDD by 18.1%. Moreover, there was a decrease in waiting list mortality between 2011 and 2012 (–4.7%). The overall waiting list mortality, too sick to transplant, and the dropout rate for other reasons were also significantly lower in Spain (15%) than in UNOS (31%) or Eurotransplant (29%; Multimedia Appendix 5).

## Discussion

### Principal Findings

In this study, we found a significant decrease in Google Trends search queries for LT in the UNOS and Eurotransplant regions. As such, public and health care providers’ levels of awareness regarding LT are decreasing alarmingly. In Spain, the leading country for transplantation, these findings were not as pronounced. Furthermore, the dropout rate in Spain was significantly lower than that in UNOS and Eurotransplant. Although the need for LT, as the only curative option for chronic liver disease, is increasing, the number of donor organs is also increasing. However, the gap between possible recipients and donors is also increasing. To close this gap, transplant and donor programs, which in part bring awareness to both the public and health care domains, may provide some improvement.

As indicated by the compelling findings presented here, the application of internet data in health care research presents a promising new field. It may further complement and extend the current data sources and foundations [43]. Approximately 90% of US citizens use the internet regularly. According to a data analysis of Pew Research Center (Washington, DC), following an ongoing rapid growth of *going online* and use of social media in the United States over the last decade, it stayed stable over the past 3 years. Comparable data are available in Europe.

*Health* and *health care* were the number 2 priorities to the US public in 2019. Internet users tend to search for health-related topics accordingly. In fact, more than 80% of all internet users look for health information on the web. Among them, 66% searched for information concerning a specific disease or medical problem (perennially the most popular purpose), and 56% were interested in a certain medical treatment or procedure. After checking emails and using search engines, looking for health topics was the third most frequent activity on the internet. Interestingly, the typical search for health information is on behalf of someone else [44]. The most popular science Facebook group boasts up to 44 million followers [45]. Limited access to internet use, especially internet search for health-related topics, has been found in minorities such as Hispanic, American Indian, and Alaska Native (PEW Research Center). This finding might in part explain the higher mortality and morbidity rates in these ethnicities compared with other ethnicities. Although health care topics on the internet are constantly rising, interest in LT has been decreasing since 2014 all over the world. This trend indicates that the topic LT is underrepresented in the web, despite a small increase seen from 2014 onward. However, the internet (eg, search engines and social media) is the largest platform for awareness programs in the field of liver disease and LT.

To date, very little is published regarding the awareness of LT. This disparity between the low search volumes of the terms relating to LT and the actual increasing number of transplantations may originate in the established low awareness campaigns of LT. Such campaigns are highly useful, as past awareness movements have proved extremely effective. For example, the *Ice Bucket Challenge* promoted awareness of amyotrophic lateral sclerosis. This activity, demonstrated by the dumping of a bucket of ice water over a person’s head, went viral in the summer of 2014 and resulted in a nearly 1000-fold increase in the Google Trends index. Subsequently, over US $220 million in funding has been raised worldwide for this rare disease. Several awareness campaigns related to other health issues in recent years have also proved immensely successful. One of the best known includes the *Red Ribbon* movement to fight HIV infection. Even a *World AIDS Day* was initiated on December 1, 1988 [46]. Other programs, such as those promoting the fight against breast cancer, were followed with significant successes in both awareness and funding. An additional notable example is the *Jade Ribbon Campaign,* which was a great success in hepatitis B virus awareness, screening, and physician follow-ups in Chinese Americans. Conversely, the term *liver disease* is strongly underrepresented in the public awareness and, in turn, the World Health Organization’s goal to eradicate hepatitis C by 2030 will most likely not be achieved. Even in well-developed countries, there is too little awareness of this disease among health care providers and the broad public [47]. The LT field was even more underrepresented. The reasons for this dearth of awareness are two-fold. The knowledge of primary care providers regarding the possibilities of LT remains insufficient. However, patients complain about a lack of information related to the nature of their disease and the potential to undergo LT.

As shown from past promotion campaigns of various other diseases, public awareness should be the key goal to increase organ donation rates. Spain’s case offers evidence of such contention. The overwhelming number of 43.4 donors per million population in the country (2016) reflects the increased level of information provided to the public regarding organ donation. Close attention to the mass media is a key point of the Spanish system and serves a preeminent way to inform the public and raise awareness. As a result of Spain’s communication policy, journalists have become extremely important in promoting organ donation. This topic is massive and continuously presented in the media. In 2016, a total of 155 Spanish media reports or news on the topic transplantation were on TV, radio, and printed press releases on the European organ donation day in October. The internet and social media were not included in the survey. The estimated audience comprised 24 million people [48]. Thus, the interest in LT in Spain has remained high over the past 16 years.

In addition, it is important to note that the number of DCDD LTs has increased significantly over the last few years in Spain. This increase in LTs, including DCDD, might reflect the success of awareness campaigns by the ONT. The ONT has established awareness programs across the country, subject to the national Spanish health ministry. Hepatologists and anesthesiologists with special training in the field of LT are representatives of transplantation programs [49]. The fruits of this work were visible in our Google Trends analysis. Specifically, there were increased search rates of the topic, *liver transplantation*, in Spain, alongside increased number of donors, transplantations, and a lower mortality rate on the waiting list. Thus, we conclude that a stable Google Trends index, compared with the global trend, reflects the success story in Spain. This underlines our hypothesis that sensitizing people for the topic could close the gap between supply and demand in LT. Furthermore, the worldwide increase of the search terms *liver transplantation* and *liver transplant* since 2014 may be because of more awareness programs, as well as an increasing number of DCDD and living donor transplants worldwide. Indeed, steps to increase awareness are underway. For example, the first National Patient Advisory Committee of America’s Liver Foundation was founded. At present, more than 50 diverse members are trained to raise awareness of the field of LT across the United States. In 2015, legislators were educated about LT and liver disease. Such discussions resulted in an annual Advocacy Day, which allowed for more awareness and an increase in search terms in the United States and worldwide.

The impact of web-based research has grown continuously in the past decade [50]. To date, Google Trends is the only unbiased approach that includes millions of users and has been widely used in economics and health issues. In economics, Google Trends data can help to improve forecasts of the current level of activity for a number of different economic time series such as automobile sales, retail sales, or unemployment [51,52]. Economists have already been at work using Google Trends to make quantitative forecasts [51,53]. Several recent research publications demonstrate that data on web searches from Google Trends can improve the accuracy of forecasts over conventional models. The use of Google data has rapidly spread in the literature to predict other economic indicators, such as analyzing their impact on stock markets and studying bond markets or their impact on commodities [54,55]. Goggle Trends and the field of infodemiology are being widely used in the field of health-related issues as well. Public attention in different fields of health care has been published recently (eg, osteoarthritis, breast cancer, or chronic inflammatory lung disease) [34,56,57]. Furthermore, infodemiology and Google Trends are used to generate awareness profiles and are suitable substitutes for classical data collection, such as surveys [50]. Thus far, Google Trends has been primarily used to monitor disease control and awareness in cancer, HIV, or stroke and also in rare diseases such as antiphospholipid syndrome or systemic lupus erythematosus [35,58-60]. Google Trends offers a wide range of capabilities, with one being the detection of success rates of awareness programs [61,62].

### Limitations

Our data indicate multiple novel aspects in the field of LT, such as those concerning donor and recipient awareness. Nonetheless, as with any study, there are some potential limitations. Data should be interpreted with caution in the context of public health and disease awareness. Rationale is 2-fold. First, there was no information about individual searches for the analyzed topics. A bias related to a high number of search queries by health care professionals, industry, or marketing agencies cannot be excluded. Second, it is to some extent elusive which search queries are summarized in the topics defined by Google Trends algorithms, as detailed information on how Google generates these data is not provided. The selection of spelling or terms might affect the results and conclusions; therefore, we chose to use more accurate spelling by native speakers and provide a detailed description of our data-gathering approach to facilitate reproducibility. Misspellings, slang words, or different accent use were considered; foreign languages (eg, English and official languages of neighboring countries) were not taken into consideration. Furthermore, some countries (eg, Hungary and Luxembourg) have a lower number of inhabitants, thus resulting in a small sample size for these countries. This may result in huge variations in Google Trends analyses over time. Another limitation may concern rural areas, as they tend to have limited internet access. Moreover, the internet use of the term *liver transplantation* is low in some countries and their official languages. The importance of accuracy in defining search queries is exemplified by searching Google Trends for the topic *immunosuppressants*. Although not specifically representing LT, immunosuppressants are associated with LT. Hence, using the query *immunosuppressant* may be useful to analyze symptom-related interest but does not sufficiently represent LT awareness. Finally, the number of studies based on Google Trends has been increasing, but so far, there is no standardized procedure for data collection. More guidance by Google is warranted to assist researchers in establishing an optimal search strategy [63].

### Conclusions

Google Trends provides a powerful tool for evaluating public interest related to LT and associated liver diseases. According to our study, interest in LT has decreased over the last decade in all investigated countries except Spain. The success story in Spain is encouraging, as it confirms that more awareness campaigns in the field of LT are needed to close the gap between increasing demand and a small supply of potential donor organs. Therefore, international awareness programs are required. In the future, the effects of awareness programs could be evaluated using Google Trends. In line with the goal of higher awareness for solid organ transplantation, Google Trends helps to collect, analyze, report, and disseminate LT-related health data. Google Trends may, therefore, not only drive change and track progress but may also help to improve programs to counteract the current lack of public LT awareness.

## Appendix

Multimedia Appendix 1 Liver transplant numbers per country and year.

Multimedia Appendix 2 Living, deceased, and donation after circulatory determination of death donors in Eurotransplant by country.

Multimedia Appendix 3 Living, deceased, and donation after circulatory determination of death donors in Eurotransplant, United Network for Organ Sharing, and Organización Nacional de Trasplantes.

Multimedia Appendix 4 Liver waiting list removal due to death, too sick to transplant, died during transplant, and others.

Multimedia Appendix 5 United Network for Organ Sharing data on death removal by ethnicity by year in percentages (%).

## Abbreviations

DCDD: donation after circulatory determination of death
LT: liver transplantation
ONT: Organización Nacional de Trasplantes
UNOS: United Network for Organ Sharing
