# Supplementary material for: Using Infodemiology Metrics to Assess Public Interest in Liver Transplantation: Google Trends Analysis
Source: J Med Internet Res. 2021 Aug 17;23(8):e21656. doi: 10.2196/21656 (PMC8408753; doi:10.2196/21656)
Supplement: Multimedia Appendix 1 [file jmir_v23i8e21656_app1.pdf]

Multimedia Appendix 1: Liver Transplant numbers per country and year

Year	US	ET	ESP	B	LUX	NL	GER	AUT	SLO	H	CRO
2004	6642	1326	1040	206	1	166	795	135	24	0	0
2005	7015	1406	1070	230	2	118	901	142	15	0	0
2006	7302	1482	1051	234	5	107	979	141	21	0	0
2007	7202	1659	1112	268	1	159	1074	121	15	0	22
2008	7000	1686	1108	230	0	131	1122	116	22	0	65
2009	6958	1718	1099	255	0	146	1065	165	22	0	65
2010	6893	1850	971	253	3	143	1173	134	34	0	113
2011	6931	1837	1137	308	9	153	1097	127	24	0	128
2012	6876	1733	1084	289	4	157	980	127	38	8	142
2013	7026	1577	1093	306	6	139	836	141	35	51	120
2014	7344	1566	1068	270	3	176	793	162	34	122	131
2015	7775	1563	1162	296	3	164	765	150	43	122	145
2016	8497	1573	1159	316	3	163	770	154	37	100	133
2017	8740	1523	1247	321	9	170	716	160	34	91	122
2018	8875	1643	1230	318	7	193	807	158	29	93	138

Abbreviations: US (United States); ET (EUROTRANSPLANT); ESP (Spain); B (Belgium); LUX (Luxembourg); NL (the Netherlands); GER (Germany), AUT (Austria); SLO (Slovenia); H (Hungary); CRO (Croatia)

This is a Multimedia Appendix to a full manuscript published in the J Med Internet Res. For full copyright and citation information see http://dx.doi.org/10.2196/jmir.21656.
