# Supplementary material for: Using Infodemiology Metrics to Assess Public Interest in Liver Transplantation: Google Trends Analysis
Source: J Med Internet Res. 2021 Aug 17;23(8):e21656. doi: 10.2196/21656 (PMC8408753; doi:10.2196/21656)
Supplement: Multimedia Appendix 2 [file jmir_v23i8e21656_app2.pdf]

Multimedia Appendix 2: Living, diseased and DCDD donors in EUROTRANSPLANT by country

Year	B living	B deceased	B DCDD	LUX deceased	NL living	NL deceased	NL DCDD	GER living	GER deceased	GER DCDD	AUT living	AUT deceased	AUT DCDD	SLO deceased	CRO living	CRO deceased	H deceased
2004	26	180	4	1	3	163	9	71	724	0	6	129	0	24	0	0	0
2005	27	203	4	2	3	115	22	88	813	0	3	139	0	15	0	0	0
2006	19	215	19	5	3	104	14	92	887	0	2	139	0	21	0	0	0
2007	27	241	20	1	3	156	16	68	1006	0	3	118	0	15	0	22	0
2008	13	217	2	0	2	129	10	62	1060	0	4	112	3	22	1	64	0
2009	25	230	41	0	3	143	28	61	1004	0	7	158	0	22	3	62	0
2010	33	220	22	3	5	138	16	96	1077	0	2	132	1	34	2	111	0
2011	37	271	43	9	10	143	36	83	1014	0	2	125	2	24	3	125	0
2012	32	257	50	4	5	152	36	80	900	0	0	127	2	38	4	138	8
2013	42	264	51	6	2	137	48	86	750	0	2	139	1	35	1	119	51
2014	40	230	51	3	3	173	47	62	731	0	6	156	0	34	1	130	122
2015	33	263	79	3	3	161	46	48	717	0	5	145	0	43	2	143	122
2016	46	270	68	3	12	151	44	62	708	1	2	152	3	37	0	133	100
2017	36	285	59	9	9	161	64	63	653	0	3	157	4	34	1	121	91
2018	33	285	69	7	12	181	68	57	750	0	7	151	8	29	1	137	93

Abbreviations: B (Belgium); LUX (Luxembourg); NL (the Netherlands); GER (Germany), AUT (Austria); SLO (Slovenia); H (Hungary); CRO (Croatia); DCDD (cardiac determination of death)

This is a Multimedia Appendix to a full manuscript published in the J Med Internet Res. For full copyright and citation information see http://dx.doi.org/10.2196/jmir.21656.
