# Supplementary material for: Using Infodemiology Metrics to Assess Public Interest in Liver Transplantation: Google Trends Analysis
Source: J Med Internet Res. 2021 Aug 17;23(8):e21656. doi: 10.2196/21656 (PMC8408753; doi:10.2196/21656)
Supplement: Multimedia Appendix 3 [file jmir_v23i8e21656_app3.pdf]

Multimedia Appendix 3: Living, diseased, and DCDD donors in EUROTRANSPLANT, UNOS, and ONT.

Year	ET living	ET deceased	ET DCDD	US living	US deceased	US DCDD	ESP living	ESP deceased	ESP DCDD
2004	106	1221	13	323	6319		0	1040	0
2005	121	1287	26	323	6692		0	1070	0
2006	116	1371	33	288	7014		0	1051	0
2007	101	1559	36	266	6936		0	1112	0
2008	82	1604	15	249	6751		0	1108	0
2009	99	1619	69	219	6739		29	1070	20
2010	138	1715	39	282	6611		20	951	18
2011	135	1711	81	247	6684		28	1109	8
2012	121	1616	88	246	6630		28	1056	17
2013	133	1450	100	252	6774		20	1073	29
2014	112	1457	98	280	7064		21	1047	32
2015	91	1475	125	359	7416		30	1132	67
2016	122	1454	116	345	8152	450	28	1131	133
2017	112	1420	127	367	8373	517	17	1230	166
2018	110	1540	145	402	8473	537	24	1206	196

Abbreviations: US (United States); ET (EUROTRANSPLANT); ESP (Spain); DCDD (cardiac determination of death)

This is a Multimedia Appendix to a full manuscript published in the J Med Internet Res. For full copyright and citation information see http://dx.doi.org/10.2196/jmir.21656.
