# Supplementary material for: Using Infodemiology Metrics to Assess Public Interest in Liver Transplantation: Google Trends Analysis
Source: J Med Internet Res. 2021 Aug 17;23(8):e21656. doi: 10.2196/21656 (PMC8408753; doi:10.2196/21656)
Supplement: Multimedia Appendix 4 [file jmir_v23i8e21656_app4.pdf]

Multimedia Appendix 4*:* Liver waiting list removal due to death, too sick to transplant, died during transplant and others.

Year	ET	ET %	US	US %	ESP	ESP %	B	B %	NL	NL %	Ger	Ger %	AUT	AUT%	H	H %	CRO	CRO %	SLO	SLO %
2004	nA	nA	3035	29.1	nA	nA	nA	nA	nA	nA	nA	nA	nA	nA	nA	nA	nA	nA	nA	nA
2005	nA	nA	3237	29.9	nA	nA	nA	nA	nA	nA	nA	nA	nA	nA	nA	nA	nA	nA	nA	nA
2006	nA	nA	3339	29.9	nA	nA	nA	nA	nA	nA	nA	nA	nA	nA	nA	nA	nA	nA	nA	nA
2007	nA	nA	3402	31.8	nA	nA	nA	nA	nA	nA	nA	nA	nA	nA	nA	nA	nA	nA	nA	nA
2008	nA	nA	3703	32	nA	nA	nA	nA	nA	nA	nA	nA	nA	nA	nA	nA	nA	nA	nA	nA
2009	nA	nA	3613	32	318	15.5	nA	nA	nA	nA	nA	nA	nA	nA	nA	nA	nA	nA	nA	nA
2010	nA	nA	3857	33.3	338	17	76	22	32	17.8	653	33	54	27.6	nA	nA	31	22.1	6	20.1
2011	nA	nA	4188	35.5	383	18.7	84	21	44	23.2	650	33.8	51	26.8	nA	nA	27	18	4	16.6
2012	950	32.4	4100	34.4	284	14.8	67	18	45	23	762	38.2	50	27.6	nA	nA	19	12.6	7	20.6
2013	866	30.4	4085	33.6	324	16.2	67	18	41	21.4	656	31.5	51	25.5	24	48	21	15.3	6	22.2
2014	715	26.4	4207	33.8	305	15.0	67	19	41	17.8	514	37.2	40	19.1	34	30.4	17	11.8	2	6
2015	756	28.9	3912	30.8	319	14.6	81	21	64	28.6	526	34.6	39	20.2	25	20.7	15	9.6	6	20
2016	792	29.6	3636	27.6	301	15.2	55	15	44	20.1	588	40.5	27	15.1	44	32.8	30	19.7	4	13.3
2017	843	31.5	3542	26.3	277	14.7	64	17	36	16.6	643	34.3	33	16.5	30	28.6	30	19.9	7	23.3
2018	722	28.9	3536	25.8	202	13.3	65	17	41	17.1	521	34.3	26	13.2	23	21.3	39	22.4	7	20.6
2019	643	26.2	3357	23.9	nA	nA	59	16	55	21.6	451	32.7	23	14.5	24	22.2	26	17.3	5	17.2
Change%		-6.2		-5.2		-2.2		-6		3.8		-0.8		-13.1		-25.8		-4.8		-2.9
median	785.9	29.3	3672	30.6	305	14.7	69	18	44	20.7	596	35	39.4	20.6	20	20.4	25.5	16.9	5.4	18

Abbreviations: US (United States); ET (EUROTRANSPLANT); ESP (Spain); B (Belgium); LUX (Luxembourg); NL (the Netherlands); GER (Germany), AUT (Austria); SLO (Slovenia); H (Hungary); CRO (Croatia); nA (not available)

This is a Multimedia Appendix to a full manuscript published in the J Med Internet Res. For full copyright and citation information see http://dx.doi.org/10.2196/jmir.21656.
