# Supplementary material for: Using Infodemiology Metrics to Assess Public Interest in Liver Transplantation: Google Trends Analysis
Source: J Med Internet Res. 2021 Aug 17;23(8):e21656. doi: 10.2196/21656 (PMC8408753; doi:10.2196/21656)
Supplement: Multimedia Appendix 5 [file jmir_v23i8e21656_app5.pdf]

Multimedia Appendix 5*:* UNOS data on death removals by ethnicity by year in percentages (%).

	2019	2018	2017	2016	2015	2014	2013	2012	2011	2010	2009	2008	2007	2006	2005	2004
**AE**	13.2	14.5	15.4	15.9	20.8	24	24.2	25.2	25.4	24.0	24.0	24.7	25.2	27.2	30	31.2
**White**	13.1	14.3	14.9	15.9	21.2	24.1	25.5	24.3	26.0	24.6	23.9	24.7	25.9	26.2	29.3	31
**Black**	11.8	11.7	13.2	12.9	16.9	20.2	17.9	19.2	19.9	18.1	16.6	20.9	21.6	21.4	27.2	25.2
**His**	15.6	17.1	19.2	18.4	24.6	29.1	26.6	33.8	28.9	26.7	33.8	29.2	27.8	37.9	38	39.5
**Asian**	11.0	16.9	15.8	14.3	12.5	16.6	13.5	21.7	18.3	20.4	17.3	19.5	16.3	21.2	23.3	23.4
**AI/AN**	13.2	19.4	14.9	30.4	21.7	31.3	30.3	39	56.8	40.5	28.6	27	35.7	46.7	30.3	23.1
**Pacific Islander**	4.5	5.2	13.3	16.7	40	16.7	5.3	87.5	29.1	30	12.5	7.7	18.2	62.5	83.3	33.3
**Multiracial**	6.8	2.6	18.2	7.0	10.4	9.8	12.8	13.3	1.7	17.5	15.6	23.5	14.8	18.2	19.5	27.8

Abbreviations: AE (all ethnicities), His (Hispanics), AI (American Indian), AN (Alaska Native)

This is a Multimedia Appendix to a full manuscript published in the J Med Internet Res. For full copyright and citation information see http://dx.doi.org/10.2196/jmir.21656.
